# MicroRNA-335 is downregulated in bladder cancer and inhibits cell growth, migration and invasion via targeting ROCK1

**DOI:** 10.3892/mmr.2022.12799

**Published:** 2022-07-26

**Authors:** Deyao Wu, Xiaobing Niu, Huixing Pan, Yunfeng Zhou, Ping Qu, Jian Zhou

Mol Med Rep 13: 4379–4285, 2016; DOI: 10.3892/mmr.2016.5055

Subsequently to the publication of this paper, an interested reader drew to the authors' attention that, in Fig. 3 on p. 4382, the ‘Invasion’ assay data for the negative control (NC) experiments for the T24 and EJ cell lines appeared to contain an overlap of data, such that they may have been derived from the same original source even though the data were purportedly intended to show the results from differently peformed experiments.

The authors have re-examined their original data, and realize that this figure was inadvertently assembled incorrectly. The revised version of Fig. 3, showing alternative data from one of the repeated experiments, is shown below. Note that this error did not significantly affect either the results or the conclusions reported in this paper, and all the authors agree to this corrigendum. Furthermore, the authors thank the Editor of *Molecular Medicine Reports* for allowing them the opportunity to publish this corrigendum, and apologize to the readership for any inconvenience caused.

## Figures and Tables

**Figure 3.**
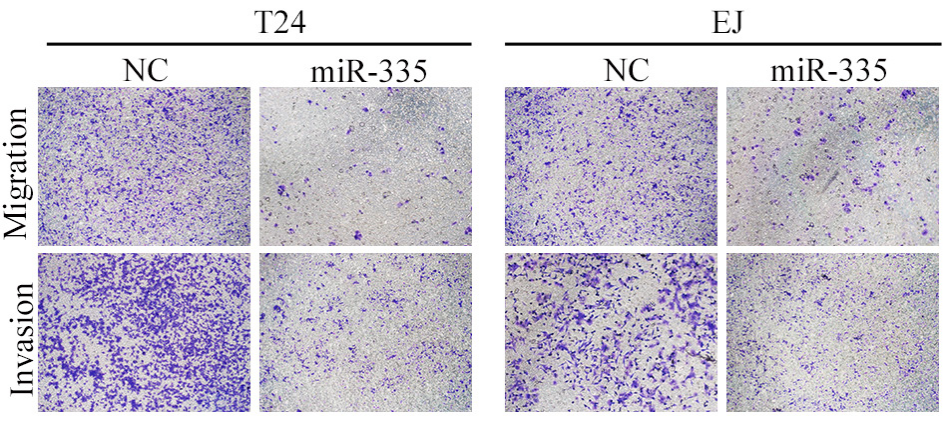
Forced expression of miR-335 suppressed cell migration and invasion in the Transwell assay. T24 and EJ cells were transfected with NC or miR-335 mimics. Cells were then incubated for 12 h for the migration assay and 24 h for the invasion assay. NC, negative control; miR-335, microRNA-335.

